# Small Left Ventricular Size Is a Risk Factor for Recurrent Pericardial Effusion after Percutaneous Drainage

**DOI:** 10.3390/jcm13092644

**Published:** 2024-04-30

**Authors:** Kousuke Akao, Teruhiko Imamura, Koichiro Kinugawa

**Affiliations:** Second Department of Internal Medicine, University of Toyama, 2630 Sugitani Toyama, Toyama 930-0194, Japan

**Keywords:** heart failure, pericarditis, pericardial drainage, hemodynamics

## Abstract

**Background:** Significant pericardial effusion requires percutaneous drainage. Some patients experience recurrent pericardial effusion following index drainage, but its risk factors remain unknown. Such knowledge should further improve the clinical management of individuals presenting with pericardial effusion for risk stratification and the construction of therapeutic and management strategies beforehand. **Methods:** Patients who underwent percutaneous drainage for pericardial effusion between 2018 and 2023 were retrospectively included and were followed for 2 years or until November 2023. Baseline factors associated with recurrent pericardial effusion that required percutaneous drainage again were investigated to identify the high-risk cohort. **Results:** A total of 39 patients (83 years on median, 28 males) were included. During the 2-year observation period, 11 patients had the primary outcome. The left ventricular end-diastolic diameter at baseline was independently associated with the primary outcome with an adjusted hazard ratio of 0.88 (95% confidence interval 0.80–0.97, *p* = 0.013) with a cutoff of 42 mm, which significantly stratified the cumulative incidence of the primary outcome (53% versus 10%, *p* = 0.011). **Conclusions:** Recurrent pericardial effusion after percutaneous drainage is not a rare phenomenon. A smaller left ventricular endo-diastolic diameter was an independent risk factor for recurrent pericardial effusion. The clinical implications of our findings in daily clinical practice should be validated in future prospective studies. Further studies are warranted to clarify the underlying causality between them.

## 1. Introduction

The pericardium, a fibroelastic sac enveloping the heart and housing a delicate layer of fluid, encounters a state of pericardial effusion when the accumulated fluid within the sac surpasses the physiological limit, typically exceeding 50 mL [1].

Pericardial effusion may manifest alongside chest discomfort or a sense of fullness [2]. However, many individuals with pericardial effusion devoid of cardiac tamponade either remain asymptomatic or exhibit indications directly linked to underlying causes (such as fever in the context of pericarditis). An extreme manifestation of pericardial effusion is cardiac tamponade, necessitating urgent intervention to alleviate the accumulation of pericardial fluid to ameliorate cardiogenic shock [3]. Cardiac tamponade arises from the accrual of pericardial fluid under pressure, impeding cardiac filling and reducing stroke volume, consequently leading to cardiogenic shock.

Pericardial effusions are often incidentally discovered during evaluations for other diseases using modalities such as chest X-ray, echocardiography, and chest-computed tomography [4]. In urgent scenarios, pericardial effusion is detected in patients experiencing hemodynamic deterioration due to cardiac tamponade.

The etiology of pericardial effusion remains idiopathic in numerous instances [5]. Primary causes of pericardial effusion encompass infection, autoimmune disorders, post-myocardial infarction conditions, post-cardiac surgery complications, chest trauma, malignancies, radiation exposure, myxedema, and certain pharmacological agents. In real-world clinical practice, we often encounter idiopathic pericardial effusion with no obviously identified etiologies.

The mortality rate associated with pericardial effusion primarily hinges upon the underlying etiologies [6]. Mortality tends to be elevated in individuals affected by fungal or bacterial pericarditis, whereas the prognosis is comparatively more favorable in those with viral pericarditis. Patients grappling with malignancy-related pericardial effusion typically exhibit poor clinical outcomes due to the advanced stage of malignancy. The clinical prognosis for individuals with idiopathic pericardial effusion remains uncertain, often observed among elderly patients who might manifest cardiac tamponade or its subclinical phases [7]. Some elderly patients have occult pericardial effusion, which impairs their activity in daily life and progresses their frailty due to limited exercise capacity related to pericardial effusion and diastolic dysfunction.

Efforts to avert the onset of cardiac tamponade or for diagnostic purposes mandate the drainage of pericardial effusion [8]. Nonetheless, the drainage of pericardial effusion is not a definite therapeutic option. A considerable number of patients experience recurrent pericardial effusion subsequent to drainage, with the risk factors for this recurrence yet to be fully elucidated. Such a recurrent pericardial effusion sometimes progress sub-clinically until it results in fatal cardiac tamponade.

A comprehensive understanding of these risk factors holds significant importance in identifying high-risk patients, necessitating vigilant monitoring and risk stratification referenced in shared decision making. In this retrospective study, we investigated the incidence of recurrent pericardial effusion and its associated risk factors.

## 2. Methods

### 2.1. Patient Selection

This study retrospectively enrolled patients who underwent the standard percutaneous drainage of pericardial effusion between the years 2018 and 2023 during their index hospitalization in a single large academic institute. The assessment of the presence and volume of pericardial effusion was conducted utilizing transthoracic echocardiography or chest-computed tomography, in principle, at the discretion of the attending cardiologists, just prior to pericardial drainage. Day 0 was designated as the date of the initial drainage procedure during the study period. Patients who did not undergo percutaneous drainage were not considered for inclusion in this study. All patients provided written informed consent, and the study protocol received approval from the institutional Ethics Committee.

### 2.2. Percutaneous Drainage

Percutaneous drainage was carefully initiated by the board-certified, well-trained attending cardiologists with an 18-gauge angiocatheter under the guidance of transthoracic echocardiography in a standard manner [9]. When the pericardial sac was entered, the sheath was advanced and the needle was withdrawn. A guidewire was then advanced through the angiocatheter, followed by a dilator and a pigtail catheter. In principle, pericardial fluid was drained fully and submitted for culture and cytological analysis.

### 2.3. Study Design

Patients were followed for two years or until November 2023 after the index drainage of pericardial effusion. The primary outcome was recurrent pericardial effusion that required percutaneous drainage again. Death and surgical pericardial fenestration were censored. Variables associated with recurrent pericardial effusion were investigated among baseline characteristics that were obtained before index drainage, as detailed below.

### 2.4. Data Collection

Demographic information, comorbidities, laboratory results, and echocardiographic findings at the time of the initial drainage were retrospectively gathered as baseline characteristics. Subsequent to discharge following the initial procedure, patients were regularly monitored by board-certified cardiologists at our institute or affiliated centers. Additional imaging studies, including chest X-rays and computed tomography scans, were conducted at each clinical visit based on the clinical judgment of attending physicians. In cases of significant pericardial effusion recurrence, the option of repeated percutaneous drainage was considered. Surgical pericardial penetration was contemplated for instances of recurrent pericardial effusion.

### 2.5. Statistical Analysis

For continuous variables, we presented data as medians with interquartile ranges (25th to 75th percentile) due to the limited sample size. Categorical variables were presented as numbers with corresponding percentages. The comparison of continuous variables between the recurrent and non-recurrent groups was conducted using the Mann–Whitney U test. Categorical variables were compared between the two groups using either the Chi-square test or Fisher’s exact test.

The primary outcome was the recurrence of pericardial effusion necessitating percutaneous drainage. Incidences of death and instances of surgical pericardial penetration were censored and not included in the primary outcome. Baseline factors associated with the primary outcome were explored using logistic regression analysis. Variables demonstrating a trend toward a difference between the recurrent and non-recurrent groups with a significance level of *p* < 0.10 were included in the univariable analysis. Variables showing a significance level of *p* < 0.05 in the univariable analysis were included in the multivariable analysis using the forced-entry method. A receiver operating characteristics analysis was performed to determine the cutoff values for variables that exhibited significance in the multivariable analysis.

All statistical analyses were conducted using SPSS version 23, and statistical significance was considered at *p*-values less than 0.05.

## 3. Results

### 3.1. Patient Selection Flow Chart

A total of 39 patients were included in this retrospective study. The median age was 83 (79, 87) years, and 28 were males. All patients were hospitalized and underwent percutaneous drainage for their clinically significant pericardial effusion by the board-certified attending cardiologists. Eighteen patients had a history of malignancy: nine had cured malignancy and the other nine patients had current active malignancy. Nineteen patients had no suspected cause of pericardial effusion (i.e., idiopathic). No patients received intravenous inotropes. A patient had a history of old tuberculosis.

The median plasma B-type natriuretic peptide was 107 (36, 310) pg/mL, and the estimated glomerular filtration rate was 54.6 (40.8, 68.9) mL/min/1.73 m^2^. The median left ventricular end-diastolic diameter (LVDD) was 41 (36, 45) mm, and the median left ventricular ejection fraction was 68% (60%, 72%).

Pericardial drainage was performed urgently in 23 patients and in a scheduled manner in the other 16 patients. The total drained fluid amount was 773 (325, 975) mL. No patients had positive results in the bacterial culture tests, except for a patient who had idiopathic pulmonary hypertension and underwent urgent pericardial drainage. *Cutibacterium acnes* was identified but was assumed to be contamination.

All procedures were successful without any procedure-related complications such as myocardial perforation, hematoma, and infection. All patients spent the index hospitalization without any clinically significant events after pericardial drainage. Seven patients received colchicine administration at the discretion of the attending cardiologists for prophylactic purposes from the index discharge. Any other pharmacological treatments were not administered to prevent recurrent pericardial effusion.

### 3.2. Recurrence of Pericardial Effusion

Patients were followed for a median of 332 (1, 730) days after the index pericardial drainage until encountering the primary outcome or study terminal. Eleven patients encountered recurrent pericardial effusion at 117 (7, 377) days after pericardial drainage. Eleven patients died and four patients underwent pericardial fenestration.

A comparison between the patients with/without recurrent pericardial effusion is displayed in Table 1. The prevalence of cured/current malignancy was not significantly different between the two groups (*p* > 0.05 for both). The prevalence of no suspected cause (i.e., idiopathic pericardial effusion) tended to be higher in patients with recurrence (*p* = 0.060). LVDD was significantly smaller and left ventricular ejection fraction was significantly higher in the recurrent group (*p* < 0.05 for both).

All drainage was performed urgently or as a scheduled procedure. The prevalence of urgent procedures tended to be lower in the recurrent group. The total fluid amount was not significantly different between the two groups (*p* = 0.11). The rate of colchicine administration at the time of index discharge tended to be lower in the recurrent group (*p* = 0.060).

### 3.3. Factors Associated with Recurrent Pericardial Effusion

In the logistic regression analysis (Table 2), various potential factors associated with recurrent pericardial effusion were examined. A smaller LVDD emerged as an independent factor linked to recurrent pericardial effusion, demonstrating an adjusted hazard ratio of 0.88 (with a 95% confidence interval of 0.80–0.97, *p* = 0.013). There was no significant correlation between the amount of pericardial effusion and baseline LVDD (*p* = 0.80, r = −0.05).

**Figure 1:**
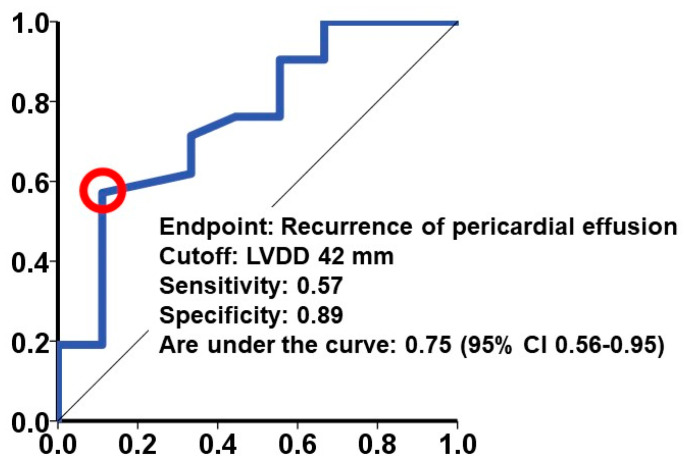
A cutoff of LVDD for the primary outcome. LVDD, left ventricular end-diastolic diameter; CI, confidence interval. Receiver operating characteristics analysis was performed to calculate a cutoff for the primary outcome defined as a recurrent pericardial effusion. A red circle represents a cutoff.

**Figure 2:**
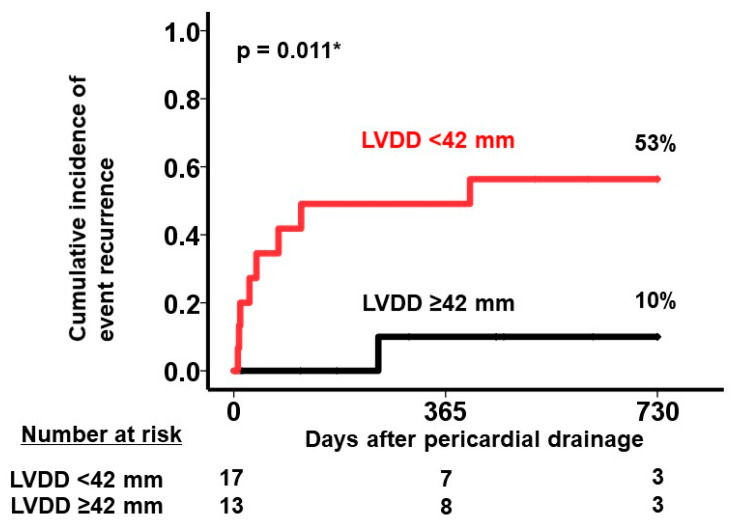
Cumulative incidence of the primary outcome during a two-year observation period after the index drainage stratified by the level of LVDD. LVDD, left ventricular end-diastolic diameter. Patients were stratified by the cutoff of LVDD. * *p* < 0.05 by log-rank test.

**Figure 3:**
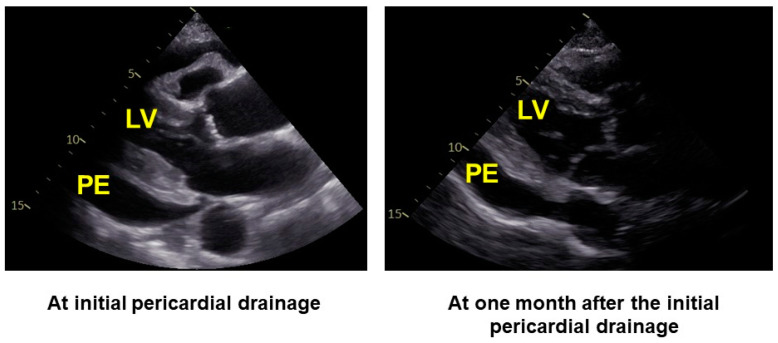
A representative patient with LVDD < 42 mm at baseline who experienced recurrent pericardial effusion. The patient was a 91-year-old man with a history of hypertension. He was admitted to our outpatient clinic complaining of bilateral leg edema and presenting 2187 pg/mL of serum N-terminal pro B-type natriuretic peptide level. A transthoracic echocardiography showed a huge amount of idiopathic pericardial effusion with stable hemodynamics. We underwent scheduled drainage for pericardial effusion without any complications. One month after the index discharge, he was again admitted to our outpatient clinic with recurrent pericardial effusion. LVDD was 33 mm on the first admission and 28 mm on the second admission. LV, left ventricle; PE, pericardial effusion.

A predictive threshold for recurrent pericardial effusion was determined at an LVDD of 42 mm, yielding a sensitivity of 0.57 and a specificity of 0.89 (Figure 1). Notably, among the 17 patients exhibiting an LVDD < 42 mm at baseline, a considerably higher cumulative incidence of recurrent pericardial effusion was observed over a 2-year observation period compared to those with values above the threshold (53% versus 10%, *p* = 0.011; Figure 2). We display a representative patient with LVDD < 42 mm at baseline who experienced recurrent pericardial effusion (Figure 3).

## 4. Discussion

In this retrospective analysis, we explored the determinants linked to recurrent pericardial effusion necessitating pericardial drainage among individuals who had undergone primary percutaneous pericardial drainage for their pericardial effusion. Among the cohort comprising 39 patients with pericardial effusion, 23 underwent urgent pericardial drainage, while the remaining 16 underwent scheduled procedures. Over the 24-month observation period subsequent to the primary drainage intervention, approximately one-third of the subjects encountered recurrent pericardial effusion, necessitating a repeated drainage procedure. A smaller LVDD at baseline, measured at the time of initial pericardial drainage, was an independent variable associated with the recurrent pericardial effusion.

### 4.1. Pericardial Effusion and Pericarditis

Pericarditis stands as one of the etiologies contributing to pericardial effusion. The diagnostic criteria for pericarditis encompass the fulfillment of at least two of the following criteria: chest pain, the presence of a pericardial friction rub, pronounced ST elevation in the electrocardiogram, and the concurrent occurrence of pericardial effusion [10]. Previous scholarly works predominantly centered on the clinical manifestations of pericarditis. Nevertheless, within clinical practice, we frequently encounter instances of pericardial effusion stemming from diverse etiologies, including idiopathic origins, not fulfilling the criteria of typical pericarditis [11]. Notably, these effusions exhibit a propensity for recurrence post pericardial drainage, posing challenges for clinicians [5]. A subset of cases may remain undetected until the manifestation of hemodynamic deterioration, because pericardia effusion often progresses without any obvious clinical signs and symptoms.

Despite these clinical nuances, there exists a dearth of literature elucidating the factors predisposing individuals to recurrent pericardial effusion. This lacuna in knowledge underscores the rationale behind our inclusive approach, incorporating all individuals with pericardial effusion, irrespective of their underlying etiologies.

A precedent investigation focusing on acute pericarditis involved a cohort of 65 patients, characterized by a median age of 65 years [12]. In our comprehensive study encompassing all individuals necessitating pericardial drainage due to pericardial effusion, the median age elevated to 83 years. This observation underscores the prevalence of pericardial effusion in the context of real-world clinical practice, particularly among the elderly demographic.

### 4.2. Recurrent Pericardial Effusion

Recurrent pericardial effusion was observed in approximately one-third of individuals afflicted with pericardial effusion, notwithstanding that nearly half of the entire patient cohort exhibited no discernible etiologies, manifesting as idiopathic pericardial effusion. The recurrence incidence aligns with prior literature documenting recurrent occurrences in the context of malignant pericardial effusion [13].

A separate investigation reported a 9.3% recurrence rate within 30 days among 553 patients [14]. Notably, of these patients, 480 initially underwent pericardial fenestration as opposed to pericardial drainage. While pericardial fenestration stands as an efficacious therapeutic measure in averting recurrent pericardial effusion, its invasiveness warrants cautious consideration, especially in individuals characterized by advanced age and multiple comorbidities mirroring our cohort. In contrast, the 30-day recurrence rate for tuberculosis-related pericardial effusion was notably higher at 30% [14], notwithstanding our lack of routine tuberculosis screening.

A conspicuous paucity of studies has systematically investigated the variables linked to recurrent pericardial effusion. Intriguingly, an idiopathic etiology devoid of specific causative factors for pericardial effusion and the absence of urgent interventions were predisposing factors for recurrent occurrences. Chronic inflammation of unknown origin, refractory to direct treatment, may contribute to a gradual escalation in pericardial effusion volume, persisting even subsequent to transient pericardial drainage [7]. An independently identified risk factor for recurrent pericardial effusion was a smaller LVDD, presumably reflective of chronic pericardial effusion inducing left ventricular remodeling. Remarkably, the quantity of drained fluid did not exhibit any discernible association with the recurrence of pericardial effusion.

### 4.3. Clinical Implication

A prior investigation underscored the efficacy of 1.2 g/day colchicine therapy in significantly mitigating the recurrence of malignant pericardial effusion [13]. In contrast, the standard 0.5 mg/day colchicine dosage, prevalent in Japan for secondary prevention [15], demonstrated ineffectuality within our study cohort, characterized by a substantial proportion of idiopathic etiologies. While pericardial fenestration represents a definitive prophylactic measure [16], its applicability extends beyond select patients, particularly among elderly individuals burdened by multiple comorbidities akin to our study population, given its inherent invasiveness. In the absence of a definitive gold standard for secondary prevention, the optimization of risk stratification emerges as paramount in enhancing clinical outcomes.

An identified independent risk factor for recurrent pericardial effusion is a smaller LVDD. Vigilant surveillance through iterative imaging studies is strongly advocated in cohorts with this heightened risk profile.

### 4.4. Limitations

This retrospective observational study is constrained by a modest sample size, potentially impacting the statistical power of the findings. Notably, certain observations that failed to attain statistical significance (*p* > 0.05) may achieve significance in more expansive investigations. Due to the limited sample size, we treated all continuous variables as non-parametric, irrespective of their distribution.

The inclusion criteria encompassed solely those patients undergoing percutaneous pericardial drainage for their pericardial effusion, excluding those who did not undergo such interventions. This selective inclusion may have omitted patients without symptoms or those facing severe systemic conditions, thus limiting the generalizability of our findings. The absence of routine screening for tuberculosis and various viral infections introduces potential gaps in our understanding, as specific causes may have been subsumed within the idiopathic etiology [11]. We tried our best to screen the underlying diseases as the cause of pericardial effusion, but any other potential etiologies may have been missed. We did not perform comprehensive cytological and histological assessments in the obtained pericardial effusion, which may have given us further insights into the underlying etiologies.

The primary endpoint was defined as recurrent pericardial effusion necessitating pericardial drainage, excluding patients who succumbed or underwent pericardial fenestration. It is conceivable that some of these excluded cases could have presented with severe recurrent pericardial effusion, but were not accounted for in our primary endpoint analysis. Noteworthy is the observation that patients with recurrent pericardial effusion exhibited a smaller LVDD despite comparable pericardial effusion volumes, suggesting potential left ventricular remodeling. However, we did not track the trajectory of LVDD post pericardial drainage and did not verify the persistent diminution of LVDD. Similarly, we do not have data on LVDD before the accumulation of pericardial effusion. These data should give us further insight into the underlying mechanisms of our findings.

## 5. Conclusions

The occurrence of recurrent pericardial effusion following percutaneous drainage is a noteworthy occurrence among patients with pericardial effusion, encompassing diverse etiologies, including idiopathic origins, in real-world daily clinical practice. An independently identified risk factor for recurrent pericardial effusion was a smaller LVDD, suggestive of potential left ventricular remodeling induced by the persistent and long-standing presence of pericardial effusion. Our findings underscore the need for further investigations to elucidate the causal relationship between LVDD and recurrent pericardial effusion, as well as to delineate the clinical implications of our observations.

## Figures and Tables

**Table 1:** Baseline characteristics stratified by the recurrence of pericardial effusion.

	Total (*n* = 39)	Recurrence (*n* = 11)	No Recurrence (*n* = 28)	*p*-Value
Demographics				
Age, years	83 (79, 87)	83 (82, 89)	76 (70, 80)	0.66
Male sex	28 (72%)	7 (64%)	21 (75%)	0.37
Body mass index, kg/m^2^	19.4 (18.7, 22.7)	18.9 (18.8, 21.6)	19.8 (19.2, 24.6)	0.65
Systolic blood pressure, mmHg	102 (92, 114)	102 (91, 115)	101 (90, 117)	0.43
Pulse rate, bpm	84 (76, 88)	83 (74, 88)	84 (75, 91)	0.32
Comorbidity				
Hypertension	19 (49%)	6 (55%)	13 (52%)	0.46
Dyslipidemia	8 (21%)	1 (9%)	7 (25%)	0.27
Diabetes mellitus	15 (39%)	3 (27%)	12 (43%)	0.37
Coronary heart disease	3 (8%)	0 (0%)	3 (11%)	0.26
Atrial fibrillation	2 (5%)	1 (9%)	1 (4%)	0.48
Heart failure	8 (21%)	3 (27%)	5 (18%)	0.51
Hemodialysis	1 (3%)	0 (0%)	1 (4%)	0.53
Collagen disease	0 (0%)	0 (0%)	0 (0%)	-
Rheumatoid arthritis	3 (8%)	1 (9%)	2 (7%)	0.84
Hypothyroidism	2 (5%)	0 (0%)	2 (7%)	0.36
Current malignancy	9 (23%)	2 (18%)	7 (25%)	0.65
Cured malignancy	9 (23%)	3 (27%)	6 (21%)	0.70
No suspected cause	19 (49%)	8 (73%)	11 (39%)	0.060
Laboratory data				
Serum albumin, g/dL	3.2 (2.9, 3.4)	3.1 (3.0, 3.2)	3.3 (2.9, 3.9)	0.43
Hemoglobin, g/dL	10.8 (10.2, 11.9)	10.6 (9.9, 11.8)	10.8 (9.9, 11.8)	0.86
Aspartate aminotransferase, IU/L	32 (26, 48)	31 (25, 47)	32 (26, 48)	0.58
Alanine aminotransferase, IU/L	41 (32, 49)	41 (31, 48)	41 (32, 49)	0.65
Serum total bilirubin, mg/dL	1.2 (0.9, 1.9)	1.2 (0.9, 1.8)	1.2 (0.9, 1.9)	0.77
Serum TSH, μIU/mL	3.4 (2.5, 4.5)	3.3 (2.4, 4.3)	3.4 (2.4, 4.6)	0.43
Plasma BNP, pg/mL	107 (36, 310)	95 (44, 290)	119 (67, 225)	0.18
Serum CRP, mg/dL	0.34 (0.28, 1.15)	0.34 (0.29, 0.34)	1.03 (0.54, 5.3)	0.50
eGFR, mL/min/1.73 m^2^	54.6 (40.8, 68.9)	63.0 (46.1, 64.6)	45.3 (40.8, 59.5)	0.61
Echocardiography data				
LVDD, mm	41 (36, 45)	39 (33, 41)	45 (45, 52)	0.028 *
LVEF, %	68 (60, 72)	70 (68, 73)	64 (49, 65)	0.008 *
Left atrial diameter, mm	33 (28, 40)	30 (29, 40)	36 (31, 38)	0.37
Pericardium data				
Urgent/scheduled drainage	23 (59%)/16 (41%)	4 (36%)/7 (64%)	19 (68%)/9 (32%)	0.072
Total protein, g/dL	4.8 (4.3, 5.3)	4.8 (3.7, 5.3)	4.8 (4.4, 5.3)	0.53
LDH, U/L	271 (159, 620)	208 (134, 450)	333 (258, 561)	0.020 *
Total fluid amount, mL	773 (325, 975)	775 (450, 950)	770 (410, 885)	0.11
Colchicine administration	7 (3%)	4 (36%)	3 (11%)	0.060

TSH, thyroid stimulation hormone; BNP, B-type natriuretic peptide; CRP, C-reactive protein; eGFR, estimated glomerular filtration rate; LVDD, left ventricular end-diastolic diameter; LVEF, left ventricular ejection fraction; LDH, lactate dehydrogenase. Continuous variables are stated as medians (25% interquartile, 75% interquartile) and were compared between the two groups using Mann–Whitney U test. Categorical variables are stated as numbers (percentage) and were compared between the two groups using Chi-square test or Fischer’s exact test as appropriate. * *p* < 0.05.

**Table 2:** Potential variables associated with the recurrence of pericardial effusion.

	Univariable Analysis		Multivariable Analysis	
	Hazard Ratio (95% CI)	*p*-Value	Hazard Ratio (95% CI)	*p*-Value
No suspected cause	2.87 (0.88–10.9)	0.12		
LVDD, mm	0.89 (0.82–0.97)	0.008 *	0.88 (0.80–0.97)	0.013 *
LVEF, %	1.09 (0.99–1.20)	0.084		
Urgent pericardial drainage	0.41 (0.12–1.39)	0.15		
Pericardial LDH, U/L	0.99 (0.99–1.01)	0.18		
Colchicine use	3.69 (1.05–13.0)	0.042 *	3.00 (0.78–11.5)	0.11

CI, confidence interval; LVDD, left ventricular end-diastolic diameter; LVEF, left ventricular ejection fraction; LDH, lactate dehydrogenase. Variables that trended towards being different between the two groups with *p* < 0.10 in Table 1 were included as potential variables. Variables significant with *p* < 0.05 in the univariable analysis were included in the multivariable analysis with a forced-entry method. * *p* < 0.05 by logistic regression analysis.

## Data Availability

Dara are available from the corresponding author upon reasonable requests.
